# Do Artifacts From Dental Implants Impair the Diagnosis of Simulated Internal Root Resorption in Cone‐Beam CT?

**DOI:** 10.1002/cre2.70373

**Published:** 2026-06-28

**Authors:** Matheus Sampaio‐Oliveira, Fernanda Bulhões Fagundes, Luiz Eduardo Marinho‐Vieira, Rubens Spin‐Neto, Matheus L. Oliveira

**Affiliations:** ^1^ Department of Oral Diagnosis, Division of Oral Radiology, Piracicaba Dental School University of Campinas (UNICAMP) Piracicaba Brazil; ^2^ Department of Dentistry and Oral Health, Section for Oral Radiology and Endodontics Aarhus University Aarhus Denmark; ^3^ Departament of Dentistry State University of Paraíba Campina Grande Brazil

**Keywords:** artifacts, dental implants, endodontics, root resorption, tomography

## Abstract

**Objectives:**

To evaluate the influence of artifacts generated by dental implants on the diagnosis of simulated internal root resorption (IRR) in cone‐beam computed tomography (CBCT), considering implant number and composition.

**Materials and Methods:**

Thirty‐five single‐rooted teeth were allocated into two groups: with simulated IRR (*n* = 15) and without IRR (*n* = 20). IRR was simulated using 37% hydrochloric acid for 3 h. Teeth were inserted into a human maxilla and scanned with two CBCT units (Axeos—Dentsply Sirona, Germany and X1—Copenhagen Imaging, Copenhagen) under three conditions: control, one adjacent implant, and two adjacent implants composed of titanium or zirconia. Three blinded observers independently assessed the CBCT volumes for IRR using a 5‐point scale. After 30 days, 15% of volumes were re‐evaluated. Sensitivity, specificity, and area under the curve (AUC) were calculated. Two‐way ANOVA with Tukey post hoc tests analyzed the effects of implant number and composition (*α* = 0.05). Weighted Kappa assessed intra‐ and inter‐observer agreement.

**Results:**

Intra‐ and inter‐observer agreements ranged from 0.84 to 0.93 and 0.83–0.84, respectively. For Axeos, two zirconia implants significantly reduced sensitivity and AUC compared with control (*p* < 0.05) and titanium implants (*p* < 0.05). For X1, two zirconia implants reduced sensitivity and AUC (*p* < 0.05), while two titanium implants reduced specificity compared with control, one titanium, and two zirconia conditions (*p* < 0.05).

**Conclusions:**

The presence of dental implants, particularly two zirconia implants, reduces the detection of IRR in CBCT.

## 1 Introduction

Internal root resorption (IRR) is an inflammatory process characterized by the loss of dentin along the root canal walls, which may progress toward the cementum due to osteoclastic activity (Patel et al. 2010; Nilsson et al. 2013). This condition has an uncertain etiology, although it is frequently associated with dental trauma (Nilsson et al. 2013). Previous studies have estimated the prevalence of IRR to range from 9.6% (Gabor et al. 2012) to 14% (Dao et al. 2023), while its incidence varies from approximately 1.2% in teeth replanted after avulsion (Souza et al. 2018) to up to 22.2% in teeth affected by intrusive luxation (de Souza et al. 2020). Teeth affected by IRR are frequently asymptomatic and often detected as incidental findings during radiographic examination (Nilsson et al. 2013; Patel et al. 2009; Kamburoğlu and Kursun 2010). When left unrecognized, the defect can extend apically, compromising the vitality of the pulp tissue beyond the resorptive site. This necrotic pulp tissue then becomes susceptible to bacterial invasion, ultimately leading to infection of the entire root canal system and the development of apical periodontitis (Patel et al. 2010). Early diagnosis is therefore essential to stop progression, preserve tooth structure, and prevent complex endodontic complications, leading to higher rates of favorable prognosis (Patel et al. 2010, 2009; Nilsson et al. 2013).

Imaging examinations play a fundamental role in the diagnosis of IRR. In this context, cone‐beam computed tomography (CBCT) is currently considered the method of choice, as it provides greater sensitivity and specificity compared to periapical radiography (Patel et al. 2009). The joint American Association of Endodontists and American Academy of Oral and Maxillofacial Radiology position statement on CBCT and the Endodontics European Society of Endodontology (Patel et al. 2023; Fayad et al. 2015) state that CBCT with limited field of view, and preferentially in high resolution, is considered the preferred imaging technique for accurately locating and distinguishing between external and internal resorptive defects, as well as for guiding treatment planning and predicting prognosis. In CBCT, IRR can appear as a well‐defined, symmetrical, round radiolucency, as well as dilations of the pulp canal (Dao et al. 2023; Patel et al. 2009; Kamburoğlu and Kursun 2010).

However, the presence of dental implants within the scanned area may compromise CBCT diagnosis due to the formation of artifacts (Schulze et al. 2011). Dental implants, whether titanium or zirconia, are increasingly used in contemporary oral rehabilitation. While CBCT is essential for implant‐based rehabilitation, artifacts from these implants can obscure adjacent teeth, potentially compromising the detection of conditions such as internal root resorption. Understanding how implant material and number influence artifact severity is therefore critical for clinicians working in implant‐supported rehabilitation.

Dental implants are capable of producing artifacts of varying intensities depending on their composition, with zirconia implants generating more pronounced artifacts than titanium implants in CBCT scans (Vasconcelos et al. 2019; Kocasarac et al. 2022). Previous studies have addressed the influence of artifacts arising from dental implants in endodontic‐related diagnostic tasks. The artifacts negatively influenced the CBCT‐based diagnosis of vertical root fracture (Candemil et al. 2021; Freitas, Vasconcelos et al. 2019), horizontal root fracture (Ruiz et al. 2024), external cervical resorption (Gonzalez‐Passos et al. 2024), and crack detection (Pinto et al. 2023), but did not negatively affect the diagnosis of external root resorption (Freitas, Nascimento et al. 2019). Regarding the influence of metallic artifacts on the diagnosis of IRR using CBCT, the presence of metallic posts in two teeth adjacent to the one presenting IRR was shown to impair the detection of this condition (Gaêta‐Araujo et al. 2020). However, the influence of artifacts arising from titanium and zirconia implants, a relatively common clinical scenario, on the diagnosis of IRR remains unanswered. Considering the clinical relevance of IRR and the importance of CBCT for its early diagnosis, allied to the fact that there is a growing number of patients with dental implants, the aim of the present study was to evaluate the impact of artifacts generated by dental implants with varying numbers and compositions on the CBCT‐based diagnosis of simulated IRR.

## 2 Materials and Methods

The research protocol described below is in accordance with the Declaration of Helsinki and was submitted for consideration to the local Research Ethics Committee (CEP‐FOP/Unicamp), and, according to the official statement No. 06/2025, it was exempted from ethical review. Informed consent was not applicable.

### 2.1 Sample Selection

A total of 35 single‐rooted teeth with completely developed roots and a single canal were included. Teeth showing pulp calcifications, internal or external resorption, history of endodontic treatment or post placement, as well as dental anomalies, were excluded. The selection criteria were verified through visual inspection and CBCT‐based assessment using the highest voxel resolution available (0.085 mm). The assessment was performed by an experienced dentomaxillofacial radiologist.

#### 2.1.1 Tooth Preparation

The selected 35 teeth were carefully cleaned and disinfected with 2% chlorhexidine. They were then sectioned lengthwise into buccal and lingual/palatal halves using a precision saw (IsoMet 1000 Buehler, Lake Bluff, IL, USA) with a compatible blade, to allow direct access to the root canal lumen.

The teeth were allocated into two groups: with IRR (*n* = 15) and without IRR (*n* = 20). A slightly larger number of teeth without IRR was included to better approximate the clinical prevalence of the condition while maintaining adequate balance between groups for statistical analyses. For the group with IRR simulation, the lesions were simulated according to a previously described protocol (Da Silveira et al. 2014). A circular piece of adhesive tape (2 mm in diameter) was initially placed in the middle of the root canal wall on both halves to delimit the area intended for IRR simulation. Each tooth was then covered with two layers of acid‐resistant nail polish applied both inside and outside. After the nail polish had dried, the adhesive tape was removed, leaving the circular area exposed for the acid application used to simulate the IRR lesion. The teeth were individually immersed in 37% hydrochloric acid for 3 h. After this period, they were rinsed under running water and dried. Then, the two halves of each tooth were reassembled with cyanoacrylate adhesive (Scotch Super Glue, 3M, Maplewood, USA), and the quality of IRR simulation was verified using CBCT at a voxel resolution of 0.085 mm.

#### 2.1.2 CBCT Image Acquisition and Export

**Figure 1:**
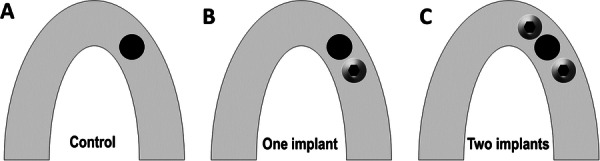
Schematic representation of the tested conditions in the maxilla (outlined arches): A. Control: only the tooth of interest (solid black circle) without a dental implant. B. One dental implant adjacent to the tooth of interest (solid black circle). C. Two dental implants adjacent to the tooth of interest (solid black circle).

The teeth were individually inserted into the socket of the left canine of a human maxilla and CBCT scans were performed under three experimental conditions: (1) control—without adjacent dental implant; (2) with one dental implant positioned adjacent to the tooth of interest, in the socket of the left maxillary first premolar; and (3) with two dental implants adjacent to the tooth of interest, one in the socket of the left maxillary first premolar and the other in the socket of the maxillary lateral incisor. All experimental conditions are illustrated in Figure 1. Additionally, in the conditions containing one or two dental implants, they were composed of either titanium or zirconia.

Image acquisition was performed using two CBCT units at their highest voxel resolution: Axeos (Dentsply Sirona, Bensheim, Germany), and X1 (Copenhagen Imaging, Copenhagen, Denmark). The Axeos unit was adjusted to operate at a 5 × 5 cm field‐of‐view, a voxel size of 0.08 mm, 6 mA, 85 kV, and an exposure time of 14.2 s. The X1 unit was adjusted to operate at a 5 × 5 cm field‐of‐view, a voxel size of 0.75 mm, 12 mA, 90 kV, and an exposure time of 11.7 s.

For CBCT acquisition, the maxilla was positioned upside down in a container filled with water to simulate soft tissue attenuation. This configuration allowed the teeth of interest to be replaced without moving the maxilla, ensuring consistent positioning across a group of acquisitions, and facilitated the insertion and removal of the dental implants when required. The container with the maxilla was supported on a tripod during CBCT acquisitions.

**Figure 2:**
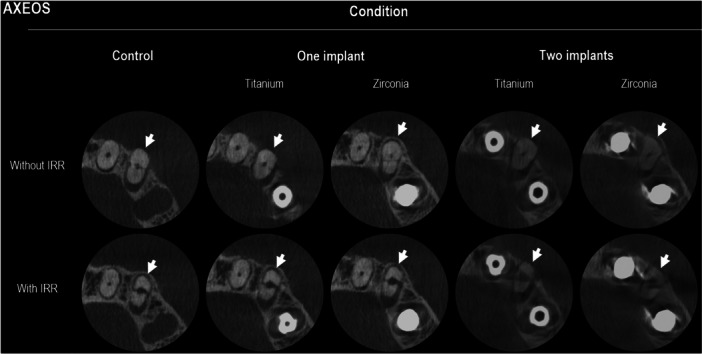
Axial sections acquired with Axeos CBCT unit showing representative teeth of interest (arrow) without and with simulated internal root resorption (IRR) as a function of the implant number (control, one‐implant, and two‐implant) and implant composition (titanium and zirconia).

**Figure 3:**
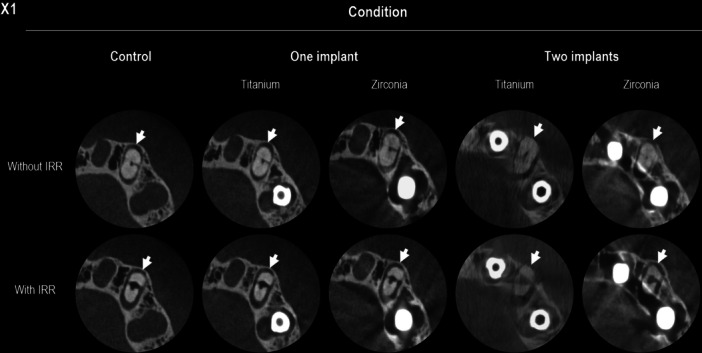
Axial sections acquired with X1 CBCT unit showing representative teeth of interest (arrow) without and with simulated internal root resorption (IRR) as a function of the implant number (control, one‐implant, and two‐implant) and implant composition (titanium and zirconia).

The resulting 350 CBCT scans {35 teeth × [1 control + (2 implant numbers × 2 implant compositions)] × 2 CBCT units} were exported in DICOM file format. Representative axial sections of each experimental condition for Axeos and X1 units are shown in Figures 2 and 3, respectively.

#### 2.1.3 Image Evaluation

CBCT image volumes were independently assessed (i.e., inspection of the axial slices) by three dentists, each with a minimum of 6 years of experience in CBCT interpretation using ImageJ software (National Institutes of Health, Maryland, USA). The observers underwent prior training and calibration and were blinded to the presence of simulated IRR and implant composition. Observer training was performed using representative CBCT volumes of teeth with and without simulated IRR, under control and different implant conditions. These volumes were used solely for training purposes and were not included in the final sample.

The observers evaluated the CBCT image volumes using an LCD monitor in a quiet environment with reduced lighting and were allowed to adjust brightness, contrast, and zoom. To minimize the risk of visual fatigue, the evaluation of CBCT scans was limited to a maximum of 35 per day. After evaluating each CBCT, the observers recorded their opinion on the presence of simulated IRR using a 5‐point scale: 1—not detectable, 2—unlikely to be present, 3—indeterminate, 4—likely to be present, 5—clearly detectable. Thirty days after completing the initial evaluations, 15% of the CBCT scans were reevaluated to test the intraobserver reproducibility.

#### 2.1.4 Data Analysis

Sensitivity, specificity, and area under the receiver operating characteristic curve (AUC) were calculated. Following exploratory and descriptive analyses, a two‐way analysis of variance (ANOVA) in a factorial design (1 control + [2 implant numbers × 2 implant compositions]) followed by Tukey's post hoc test, was conducted to evaluate the effects of implant number and composition on IRR diagnostic performance through pairwise comparisons. In addition, to facilitate understanding on how the outcomes may affect clinical decision‐making, the percentage of misdiagnosed cases for each experimental condition was calculated and classified as overdiagnosis (false‐positive cases, i.e., IRR suggested when not present) or underdiagnosis (false‐negative cases, i.e., a present IRR was missed). The significance level was set at 5% (*α* = 0.05), and all the statistical analyses were conducted using IBM SPSS Statistics for Windows (IBM Corp., Armonk, NY, USA). Intra‐ and inter‐observer agreements were assessed using weighted Kappa and interpreted according to the criteria proposed by Landis and Koch (1977).

A post hoc power analysis was performed using G*Power (version 3.1.9.7, Heinrich Heine University, Düsseldorf, Germany). Based on the smallest mean difference observed between experimental conditions, the corresponding pooled standard deviation, and the number of CBCT volumes per group included in the two‐way analysis of variance (ANOVA), the achieved statistical power was 0.83 at a significance level of 0.05.

The data supporting the findings of this study are available from the corresponding author upon request. This study was conducted in accordance with the STARD (Standards for Reporting Diagnostic Accuracy Studies) guidelines.

## 3 Results

Intra‐ and inter‐observer agreements ranged from 0.84 to 0.93 and from 0.83 to 0.84, respectively, and were considered almost perfect based on Landis and Koch's (1977) criteria.

As shown in Table 1, for the Axeos CBCT unit, sensitivity ranged from 0.53 to 1.00, specificity from 0.93 to 1.00, and AUC from 0.53 to 1.00, with a tendency for higher values associated with the titanium implants. The presence of two zirconia implants resulted in a significant reduction in both sensitivity and AUC compared with the control group (*p* < 0.05). In addition, in the presence of two implants, sensitivity was significantly lower for zirconia than for titanium (*p* < 0.05). In the absence of dental implants, 4% of cases were misdiagnosed (0% overdiagnosed and 4% underdiagnosed). When a single titanium implant was present, all cases were correctly diagnosed. With two titanium implants, 16% of cases were misdiagnosed (3% overdiagnosed and 13% underdiagnosed). In the presence of a zirconia implant, 29% of cases were misdiagnosed (7% overdiagnosed and 22% underdiagnosed). Finally, with two zirconia implants, misdiagnosis increased to 52% (5% overdiagnosed and 47% underdiagnosed).

**Table 1:** Mean values (standard deviation) of sensitivity, specificity, and area under the curve (AUC) as a function of the implant number and composition obtained with the Axeos CBCT unit.

	Control	Implant composition	Implant number	
			One	Two
Sensitivity	0.96 (0.77)	Titanium Zirconia Titanium Zirconia Titanium Zirconia	1.00 (0.00)	0.87 (0.69)
			0.78 (0.17)	0.53 (0.66)^a^, ^b^
Specificity	1.00 (0.00)		1.00 (0.00)	0.97 (0.58)
			0.93 (0.58)	0.95 (0.87)
AUC	0.98 (0.04)		1.00 (0.00)	0.71 (0.12)
			0.86 (0.07)	0.53 (0.07)^a^

^a^ Significantly lower than control (*p* < 0.05).

^b^ Significantly lower than two titanium implants (*p* < 0.05).

**Figure 4:**
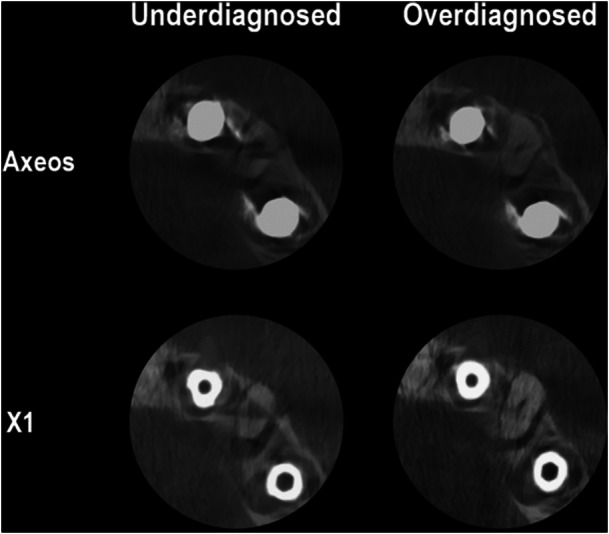
Representative axial slices illustrating underdiagnosed (false‐negative) and overdiagnosed (false‐positive) observations for each CBCT unit. The examples were selected from cases in which all evaluators provided incorrect responses. For the Axeos unit, the images correspond to the condition with two zirconia implants, whereas for the X1 unit, the images correspond to the condition with two titanium implants.

As shown in Table 2, for the X1 CBCT unit, sensitivity ranged from 0.69 to 1.00, specificity from 0.95 to 1.00, and AUC from 0.87 to 1.00, also with a tendency for higher values associated with the titanium implants. The presence of two titanium implants resulted in lower specificity compared with the control group, one titanium implant, and two zirconia implants (*p* < 0.05). The presence of two zirconia implants significantly reduced sensitivity compared with the control group (*p* < 0.05). The presence of two titanium or zirconia implants was associated with lower AUC values compared with the control and in the presence of one implant of the same composition (*p* < 0.05). In the absence of dental implants and with a single titanium implant, all cases were correctly diagnosed (0% misdiagnosis). In the presence of a zirconia implant, 6% of cases were misdiagnosed (2% overdiagnosed and 4% underdiagnosed). With two titanium implants, 9% of cases were misdiagnosed (5% overdiagnosed and 4% underdiagnosed). Finally, with two zirconia implants, 31% of the cases were misdiagnosed (0% overdiagnosed and 31% underdiagnosed). Representative axial slices of the under (false negative)‐ and overdiagnosed (false positive) observations for each CBCT unit, selected from cases in which all evaluators agreed on the same response, are shown in Figure 4. Representative axial slices of the under (false negative)‐ and overdiagnosed (false positive) observations for each CBCT unit, selected from cases in which all evaluators agreed on the same response, are shown in Figure 4. The cases were selected within the same CBCT unit from conditions in which all evaluators provided incorrect responses.

**Table 2:** Mean values (standard deviation) of sensitivity, specificity, and area under the curve (AUC) values as a function of the implant number and composition obtained with the X1 CBCT unit.

	Control	Implant composition	Implant number	
			One	Two
Sensitivity	1.00 (0.00)	Titanium Zirconia Titanium Zirconia Titanium Zirconia	1.00 (0.00)	0.96 (0.39)
			0.96 (0.39)	0.69 (1.02)^a^, ^b^
Specificity	1.00 (0.00)		1.00 (0.00)	0.95 (0.03)^a^, ^c^, ^d^
			0.98 (0.29)	1.00 (0.00)
AUC	1.00 (0.00)		0.99 (0.02)	0.95 (0.03)^a^, ^d^
			0.97 (0.01)	0.87 (0.03)^a^, ^d^

^a^ Significantly lower than control (*p* < 0.05).

^b^ Significantly lower than two titanium implants (*p* < 0.05).

^c^ Significantly lower than two zirconia implants (*p* < 0.05).

^d^ Significantly lower than one implant of the same composition (*p* < 0.05).

## 4 Discussion

The present study evaluated the impact of artifacts from titanium and zirconia dental implants on CBCT‐based diagnosis of simulated internal root resorption (IRR). Early detection of IRR is critical for tooth prognosis, as delayed diagnosis may allow lesion progression and increase the risk of dentinal perforation (Patel et al. 2010; Nilsson et al. 2013; Gabor et al. 2012; Souza et al. 2018). The results showed that dental implants, particularly two zirconia implants, markedly reduced diagnostic performance. Although CBCT is widely used in endodontics, artifacts may compromise image quality and diagnostic accuracy (Kamburoğlu and Kursun 2010). By assessing the influence of implant number and composition, this study highlights clinical situations that may challenge IRR detection in practice. According to the European Society of Endodontology, IRR may present as internal surface resorption, tunneling defects, internal inflammatory root resorption with balloon‐like enlargement of the canal space, or internal replacement resorption with irregular radiolucency (Patel et al. 2023). The simulation method used in this study produced features resembling inflammatory or tunneling IRR.

Dental implants are widely used in oral rehabilitation, and their associated artifacts are frequently encountered in CBCT scans. It is therefore important to assess whether these artifacts compromise diagnostic accuracy in the detection of IRR. In the present study, zirconia dental implants impaired diagnostic performance more than titanium dental implants, consistent with previous studies showing that zirconia generates more pronounced artifacts, likely due to its higher atomic number (Vasconcelos et al. 2019). Materials with higher atomic numbers preferentially absorb lower‐energy X‐ray photons, leading to beam hardening and a higher proportion of high‐energy photons reaching the detector, which may contribute to darker gray values in the reconstructed image (Schulze et al. 2011). This process increases beam hardening and streak artifacts in CBCT images, hindering visualization of adjacent dental structures (Schulze et al. 2011). In the present study, two zirconia dental implants reduced sensitivity and AUC values regardless of the CBCT unit. In addition to composition, the number of dental implants also influenced diagnostic performance. For the X1 unit, both titanium and zirconia dental implants in the two‐implant condition showed lower AUC values compared with the control and one‐implant conditions, suggesting a cumulative artifact effect. Clinically, these findings indicate that cases involving adjacent dental implants may represent a challenging scenario for CBCT‐based IRR detection.

This study further examined the frequency of misdiagnosed cases under different experimental conditions. For both CBCT units, the absence of dental implants resulted in no misdiagnosis, while the presence of a single titanium implant caused a small number of underdiagnosed cases in the Axeos unit. Zirconia implants, particularly when two were present, substantially increased underdiagnosis in both units. For the Axeos unit, two zirconia implants led to 47% of cases being underdiagnosed, whereas for the X1 unit, the same condition resulted in 31% underdiagnosis. These findings are particularly relevant for the diagnosis of IRR, where early detection is critical for tooth prognosis and for preventing dentinal perforations (Patel et al. 2010; Nilsson et al. 2013; Gabor et al. 2012; Souza et al. 2018). Careful interpretation of CBCT images, especially in the presence of dental implants, is therefore essential to minimize the risk of underdiagnosis and preserve tooth structure.

To the best of the authors’ knowledge, this is the first study to evaluate the influence of dental implants on the CBCT‐based diagnosis of simulated IRR. Previous studies investigating teeth restored with metal posts reported lower AUC and sensitivity values when a tooth with IRR was positioned between two teeth with posts (Gaêta‐Araujo et al. 2020). Similarly, in the present study, the presence of two adjacent implants reduced both AUC and sensitivity, suggesting that dental implant‐related artifacts can compromise the diagnostic accuracy of CBCT for detecting simulated IRR. This investigation focused on dental implants adjacent to the tooth of interest, a clinically common situation in which artifacts may directly interfere with image interpretation. Compared with intracanal posts, implants are more robust structures positioned at a depth similar to the root surface. Because artifacts usually occur in the same axial plane as the object generating them, dental implant‐related artifacts are more likely to affect the visualization of root alterations than those produced by coronal restorations or intracanal posts.

Metallic objects located outside the field of view but within the path of the primary X‐ray beam (i.e., in the exomass) may generate artifacts capable of degrading CBCT image quality and potentially affecting diagnostic accuracy (Schulze et al. 2011; Gaêta‐Araujo et al. 2020; Da Silveira et al. 2014; Landis and Koch 1977). In addition, motion artifacts caused by involuntary movements such as breathing or slight head shifts may produce double contours and image blurring, reducing diagnostic reliability (23). These artifacts may also interact, as the impact of exomass‐related artifacts can be aggravated by patient motion during scanning (Gaêta‐Araujo et al. 2020). Therefore, the present study represents a best‐case scenario, as other types of artifacts present in clinical situations could further influence diagnostic outcomes.

Differences between the two CBCT units tested should also be highlighted. While both devices showed a decrease in diagnostic performance in the presence of artifacts, the magnitude and affected parameters varied. This variation may be attributed to differences in hardware design, image reconstruction algorithms, and exposure parameters (voxel size, mA, kV, exposure time), all of which are known to influence artifact expression (Freitas, Vasconcelos et al. 2019; Ruiz et al. 2024; Gonzalez‐Passos et al. 2024). Such variability across CBCT units has also been confirmed in a previous study, where subjective image quality was shown to differ significantly among devices, with some scanners being more robust against artifacts than others, regardless of scanning protocol (Pinto et al. 2023). Therefore, clinicians should be aware that the impact of artifacts may not be uniform across different CBCT units.

The ex vivo design of this study allowed standardization and reproducibility while approximating clinical conditions. The method used to create IRR defects, based on acid demineralization rather than mechanical drilling, has been previously validated and produces image features closer to those observed clinically (Gaêta‐Araujo et al. 2020; Da Silveira et al. 2014). This realistic simulation, combined with the observers’ expertise, likely contributed to the high diagnostic performance observed. Clinically, the decreased sensitivity associated with zirconia implants is concerning, as it increases the likelihood of false‐negative diagnoses. Because IRR is often asymptomatic and requires early detection to prevent extensive tooth destruction and endodontic complications, adjacent implants may hinder timely diagnosis and potentially compromise treatment outcomes (Patel et al. 2010, 2009).

Future research should further explore the effectiveness of artifact reduction strategies, such as metal artifact reduction tools and artificial intelligence approaches to determine their potential to improve CBCT‐based diagnostic accuracy of IRR. Additionally, studies assessing the influence of different acquisition parameters, such as voxel size, mA, kV, and low‐dose CBCT protocols in the diagnosis of IRR are also recommended.

In conclusion, the presence of dental implants, particularly two zirconia implants, reduces the detection of internal root resorption on CBCT. Therefore, interpretations should be made cautiously to minimize the risk of false negative findings.

## Author Contributions

**Matheus Sampaio‐Oliveira** contributed to conceptualization, data curation, formal analysis, investigation, methodology, validation, visualization, and writing – original draft. **Fernanda Bulhões Fagundes** was involved in conceptualization, data curation, formal analysis, investigation, methodology, supervision, visualization, and writing – review and editing. **Luiz Eduardo Marinho‐Vieira** contributed to data curation, formal analysis, validation, visualization, and writing – review and editing. **Rubens Spin‐Neto**, contributed to conceptualization, data curation, formal analysis, investigation, methodology, supervision, validation, visualization, and writing – review and editing. **Matheus L. Oliveira** contributed to conceptualization, data curation, formal analysis, investigation, methodology, supervision, validation, visualization, and writing – review and editing.

## Conflicts of Interest

The authors declare no conflicts of interest.

## Supporting information

Supporting File

## Data Availability

The data that support the findings of this study are available from the corresponding author upon reasonable request.
