# Supplementary material for: Do Artifacts From Dental Implants Impair the Diagnosis of Simulated Internal Root Resorption in Cone‐Beam CT?
Source: Clin Exp Dent Res. 2026 Jun 28;12(3):e70373. doi: 10.1002/cre2.70373 (PMC13310529; doi:10.1002/cre2.70373)
Supplement: Supplementary file 1 — Supporting File [file CRE2-12-e70373-s001.pdf]

Faculdade de Odontologia de Piracicaba
UNICAMP

OF. CEP/FOP N. º 06/2025

Piracicaba, 2 June 2025

To the esteemed Dr Matheus Sampaio de Oliveira
Doctoral Candidate in the Postgraduate Programme in Dental Radiology
Piracicaba Dental School – University of Campinas (FOP/UNICAMP)

Following an analysis of the documentation submitted to the Research Ethics Committee of FOP (CEP-FOP), including the research project entitled *"Assessment of the Influence of Artefacts from Dental Implants on the Diagnosis of Internal Root Resorption in Cone Beam Computed Tomography"*, authored by Matheus Sampaio de Oliveira (Doctoral Candidate in the Postgraduate Programme in Dental Radiology, at FOP-UNICAMP), Fernanda Bulhões Fagundes (Doctoral Candidate in the same programme), Rubens Spin-Neto (Faculty Member at Aarhus University, Denmark), and Matheus Lima de Oliveira (Faculty Member in Dental Radiology, at FOP-UNICAMP), I hereby inform you that, based on the information provided in the submitted materials, the project does not, in principle, require submission for review via the Plataforma Brasil to a Research Ethics Committee involving human subjects in Brazil.

The information exchanged via email between 1 and 2 June 2025, as well as the attached file (*Projeto_Dinamarca.pdf*), indicates that the research will be conducted entirely in Denmark, with no involvement of participants, data, or biological samples of Brazilian origin. Please be advised that the information provided regarding this project will be archived by CEP-FOP-UNICAMP for a period of five years. We remain at your disposal should you require any further information.

Prof Jacks Jorge Junior
Coordinator

Av. Limeira, 901 - Bairro Areão - PIRACICABA /SP - CEP 13.414.903 - Caixa Postal 52
Fone/Fax: (19) 2106.5349 - E-Mail: cep@fop.unicamp.br
Home Page: http://www.fop.unicamp.br/cep

Documento assinado. Verificar autenticidade em sigad.unicamp.br/verifica
Informar código 80449854 01CA462E 9145111D CFF350FE

Documento assinado eletronicamente por JACKS JORGE JUNIOR, Professor Associado III, em 02/06/2025, às 17:45 horas, conforme Art. 10 § 2º da MP 2.200/2001 e Art. 1º da Resolução GR 54/2017.

A autenticidade do documento pode ser conferida no site: sigad.unicamp.br/verifica, informando o código verificador: 80449854 01CA462E 9145111D CFF350FE

UNICAMP
SIGAD
